# Endorsement of Dr. Wiley

**Published:** 1911-08

**Authors:** 


### Endorsement of Dr. Wiley.

Over three hundred registrations out of a possible eight hundred at the Ohio State Eclectic Medical Association meeting evidenced the renewed interest taken by eclectics in society affairs. The meeting was held at Cedar Point, July 18-20, 1911. The following self-explanatory resolution was presented by Prof. John Uri Lloyd, and was unanimously adopted:

*"To Hon. William H. Taft, President of the United States:*

"The Ohio Eclectic Medical Association, now in session at Cedar Point, represents eight hundred Ohio physicians, and respectfully petitions your excellency for the retention of Dr. Harvey W. Wiley as chief chemist of the Department of Agriculture. His fearless and unbiased enforcement of the pure food and drug law has already swept out of existence a multitude of depraved, corrupted and adulterated drugs, foods and compounds, thus aiding physicians and pharmacists in caring for the people's health.

"Prof. H. H. Rusby of New York is a scientist above reproach, a botanist of international reputation and a world-wide authority on medical plants. His expert services in the New York custom house should not be lost to the Department of Agriculture.

"We sincerely hope that these men will not be disturbed in their respective offices."

The attempt by food adulterators to rid the Department of Agriculture of efficient and fearless officials is rousing a storm of protest.—*Lancet-Clinic.*

Seattle (Wash.) passed an ordinance making the ventilating, disinfecting and cleaning of street cars compulsory. We quote Sections 2 and 3 verbatim:

"The interior of all street railway cars and all parts of such cars as are used by the general public in entering or leaving such cars, shall be thoroughly cleansed and dusted at least once each day.

"At least once each week the interior, platforms and hand rails of all street railway cars shall be cleansed by flushing and scrubbing with a disinfecting fluid composed of a solution of bichloride of mercury, or other suitable disinfecting fluid of such strength as to destroy all germ life. All strap hangers, seats and windows shall also once a week be thoroughly cleansed and disinfected. All window boxes must be cleared of all accumulations and must be sprayed with a disinfecting fluid or otherwise rendered free from germ life."

It seems rather a sad commentary on modern business methods that ordinary cleanliness can only be obtained by legislative enactment. "Big business" will some day come to a realizing sense of its obligations to the public. Reaction will set in, and the pendulum will swing from the present era of intense individualism to just as undesirable collectivism with an autocratic, fraternalistic bureaucracy.—*Lancet-Clinic.*

